# Increased SIRT1 Concentration Following Four Years of Selenium and Q_10_ Intervention Associated with Reduced Cardiovascular Mortality at 10-Year Follow-Up—Sub-Study of a Previous Prospective Double-Blind Placebo-Controlled Randomized Clinical Trial

**DOI:** 10.3390/antiox12030759

**Published:** 2023-03-21

**Authors:** Trine Baur Opstad, Jan Alexander, Jan Aaseth, Anders Larsson, Ingebjørg Seljeflot, Urban Alehagen

**Affiliations:** 1 Center for Clinical Heart Research, Department of Cardiology, Oslo University Hospital Ullevål, P.O. Box 4950, Nydalen, N-0424 Oslo, Norway; 2 Faculty of Medicine, University of Oslo, N-0370 Oslo, Norway; 3 Norwegian Institute of Public Health, P.O. Box 222, Skøyen, N-0213 Oslo, Norway; 4 Department of Research, Innlandet Hospital Trust, P.O. Box 104, N-2381 Brumunddal, Norway; 5 Faculty of Health and Social Sciences, Inland Norway University of Applied Sciences, N-2624 Lillehammer, Norway; 6 Department of Medical Sciences, Uppsala University, SE-751 85 Uppsala, Sweden; 7 Division of Cardiovascular Medicine, Department of Medical and Health Sciences, Linköping University, SE-581 85 Linköping, Sweden

**Keywords:** sirtuin1, selenium, coenzyme Q_10_, intervention, cardiovascular mortality

## Abstract

*Background*: Selenium and coenzyme Q_10_ (SeQ_10_) possess antioxidant and anti-inflammatory properties, potentially mediated via Sirtuin1 (SIRT1). We aimed to investigate the influence of a SeQ_10_ intervention on SIRT1 concentration, with potential interactions with microRNAs. *Methods*: In this sub-study of a prospective double-blind placebo-controlled clinical trial, healthy subjects (mean age 76 years) were randomized to receive an active treatment (*n* = 165, combined 200 µg/day of Se and 200 mg/day of Q_10_) or a placebo (*n* = 161). SIRT1 concentration and microRNAs were measured with ELISA and PCR, respectively. *Results*: After four years, SIRT1 concentration was increased in the active treatment group, with mean (SD) ng/mL of 469 (436) vs. 252 (162), *p* < 0.001, and decreased in the placebo group, 190 (186) vs. 269 (172), *p* = 0.002, and the differences between the groups were significant (*p* = 0.006, adjusted). Those who suffered CV death during a 10-year follow-up (*n* = 25 and *n* = 52 in the active treatment and placebo groups, respectively) had significantly lower baseline SIRT1 concentrations compared to the survivors (*p* < 0.001). MiR-130a-3p was significantly downregulated during the intervention and correlated inversely with SIRT1 at baseline (r = −0.466, *p* = 0.007). *Conclusion*: The increased SIRT1 concentration after the SeQ_10_ intervention associated with reduced CV mortality, partly mediated via miR-1303a-3p, suggests that SIRT1 is an additional mediator of the intervention, preventing vascular ageing.

## 1. Introduction

It is now well accepted that an optimal supply of the essential trace element selenium (Se) has multiple health-promoting benefits, and supplementation may be beneficial in subjects with low Se levels [1,2,3,4]. With its anti-oxidative and anti-inflammatory effects afforded by a number of selenoproteins, Se has been shown to reduce the harm mediated by reactive oxygen species and to reduce inflammation [2,5,6]. Coenzyme Q_10_ (ubiquonone) is another known regulator of oxidative stress. Coenzyme Q_10_ is primarily present in the mitochondria and is a component of the electron transport chain but also acts as a lipophilic antioxidant elsewhere. Coenzyme Q_10_ supplementation has also been shown to be specifically beneficial in the elderly, as the endogenous production of this enzyme declines with age [7,8,9]. There is an important relationship between Se and coenzyme Q_10,_ as the reduction of ubiquinone to its active form ubiquinol is dependent on the selenoenzyme thioredoxin reductase. Their syntheses are both dependent on a functional mevalonate pathway [10]. The main project was a prospective double-blind randomized placebo-controlled study where an elderly community-living population was given combined Se and coenzyme Q_10_ or a placebo as a dietary supplement for four years. After a median follow-up time of 5.5 years, a significantly reduced cardiovascular (CV) mortality was observed, with a significantly lower concentration of the natriuretic peptide N-terminal proBNP as a sign of less cardiac wall tension, and a significantly better cardiac function [11]. As the results were surprisingly positive, we wanted to follow our study population also after the intervention was terminated and we could report still significantly reduced CV mortality after 10 and 12 years [12,13]. We also reported beneficial effects on inflammatory markers [14,15], oxidative stress [16], endothelial dysfunction [17] and telomere attrition [18] in elderly Swedish citizens with low Se levels.

The beneficial properties of Se seem partly to be mediated by the sirtuin system [2,19], which is a class of nicotinamide adenine dinucleotide (NAD^+^)-dependent deacetylases involved in metabolism, ageing and longevity [20]. The family of sirtuins contains seven enzymes, of which sirtuin1 (SIRT1) is the most investigated. SIRT1 localizes in the nucleus and cytoplasm and is implicated in the regulation of gene transcription [21]. SIRT1 was previously shown to target the nuclear factor kappa-light-chain-enhancer of activated B-cells (NF-κB) [22], the forkhead box O class (FOXO) transcription factor and p53, thus possessing anti-inflammatory and anti-oxidative properties. It also contributes to genome stability [23]. In the elderly, SIRT1 expression and activity are decreased in several tissues and organs, including the CV system, and the lack of SIRT1 has been suggested as a mediator of CV disease (CVD) [24].

Knowledge on the potential effects of Se on SIRT1 is sparse. The downregulation of sirtuins was observed in peripheral blood mononuclear cells in CVD patients with Se deficiency [19]. Recently, increased liver expression of SIRT1 was reported in binge drinking rats when supplemented with Se [25]. Knowledge on the effect of coenzyme Q_10_ on SIRT1 is also limited, although the Q_10_ status seems to influence SIRT1 activity [26], as the decline in hepatic SIRT1 expression in diabetic rats was reversed by coenzyme Q_10_ administration [27].

A selenium and coenzyme Q_10_ intervention also seemed to have regulatory effects on microRNAs, which are small, non-coding RNAs, functioning as regulatory molecules in post-transcriptional mRNA and protein translation. Our group has recently reported on significant changes in several circulating microRNAs after such supplementation [28], of which some have been reported to target SIRT1 mRNA [29].

The impact of Se and coenzyme Q_10_ supplementation on SIRT1 needs to be further explored in a clinical experimental setting. We therefore aimed to investigate the potential effects on circulating SIRT1 of a Se and coenzyme Q_10_ intervention in elderly Swedish citizens with low levels of Se and any influence this potential SIRT1 change may have on the risk of CV mortality. As microRNAs appear to be important regulators of SIRT1 expression, we also aimed to seek associations between SIRT1 and certain microRNAs, as well as markers of inflammation and endothelial function.

## 2. Materials and Methods

### 2.1. Study Population

**Figure 1:**
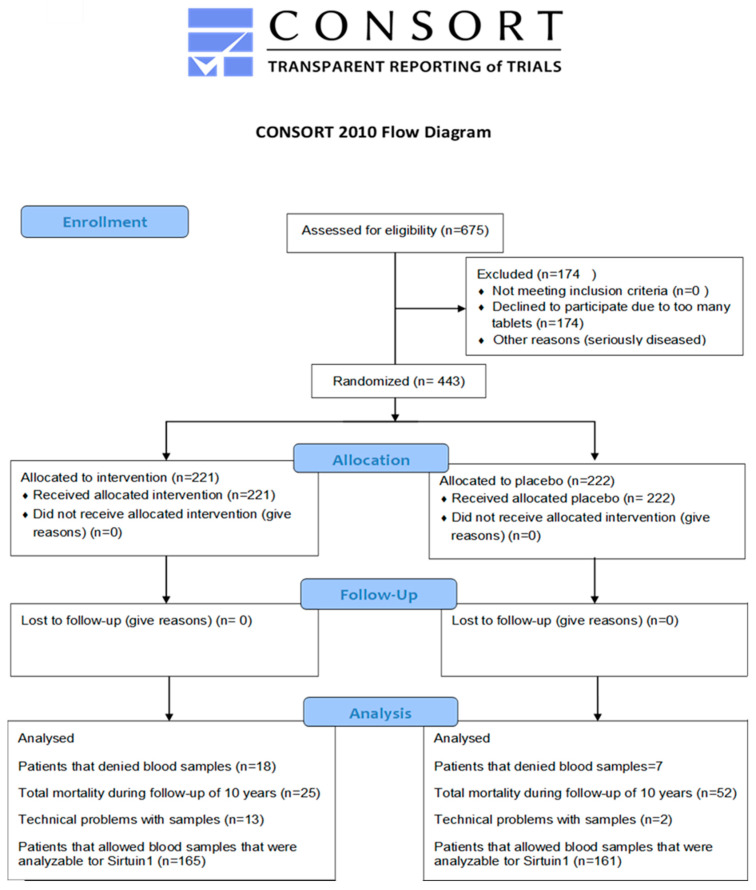
CONSORT 2010 flow diagram.

The present investigation is a sub-study of a previous prospective double-blind randomized placebo-controlled single-center trial performed between 2003 and 2010 in the south-east of Sweden [12]. The study included 443 subjects recruited from a rural municipality of 10,300 inhabitants, and the inclusion criterion was being aged >69 years. Exclusion criteria were recent myocardial infarction, planned CV operative procedure within four weeks, serious disease that substantially reduced survival, or expectation that the participant could not cooperate for the full intervention period [12]. The intervention period was four years; 221 participants received the active supplement, and 222 participants received a placebo. The participants were given either a combination of supplements consisting of yeast tablets containing 200 µg/day of organic Se (SelenoPrecise 100 µg, Pharma Nord ApS, Vejle, Denmark, twice daily) and capsules containing 200 mg/day of coenzyme Q_10_ (Bio-Quinon 100 mg twice daily, Pharma Nord ApS, Vejle, Denmark) or placebo tablets/capsules. The SelenoPrecise^®^ 100 µg tablet is approved in Denmark as a pharmaceutical drug by the Danish Medicines Agency, and the Q_10_ capsules were identical to Myoqinon^®^ (Pharma Nord ApS, Vejle, Denmark), which is a pharmaceutical drug authorized in European Union Member States (No. OGYI 11494-2010). The placebo tablets for Se and coenzyme Q_10_ contained bakers’ yeast only and 500 mg of vegetable oil with 3.1 mg of added vitamin E, respectively. All participants were supplemented for 48 months, and the non-consumed study medications were returned and counted as a measure of compliance. In the present sub-study, samples for SIRT1 analysis at inclusion were available from 326 individuals, of whom 165 received the active treatment, and 161 received the placebo (Figure 1). At 48 months, serum for the SIRT1 analysis was available from 103 and 77 subjects, respectively.

At inclusion, all study participants were clinically examined using the assessment based on the New York Heart Association functional class (NYHA class) and with an electrocardiogram and Doppler echocardiography.

The study was registered at Clinicaltrials.gov and has the identifier NCT01443780.

### 2.2. Blood Sampling

Blood samples were collected under fasting conditions both at inclusion and after 48 months. Routine analyses were carried out by conventional methods. Serum was prepared by centrifugation within 1 h from blood collection at 2.500× *g* for 10 min and stored at −70 °C until SIRT1 determination. Pre-chilled ethylenediaminetetraacetic acid (EDTA) vials were centrifuged at 3000× *g*, +4 °C, and EDTA plasma was frozen at −70 °C for the measurement of Se concentration, previously analyzed by inductively coupled plasma mass spectrometry (ICP-MS) [3].

### 2.3. SIRT1 Analysis

The Human SIRT1 ELISA kit from LSBio LifeSpan BioSciences lnc., Seattle, WA, USA, was used for the SIRT1 analysis, performed in serum at baseline and after 48 months. The samples collected at both time points from the same individual were analyzed on the same ELISA plate to minimize assay variability between runs. SIRT1 was successfully measured in all available samples, and the inter-assay coefficient of variation was 13.5%.

### 2.4. MicroRNA Analysis

The data on microRNA profiling after Se/Q_10_ intervention were retrieved from previous analyses [28]. In short, 25 participants from each randomized group in the main Se/coenzyme Q_10_ intervention trial [12] were evaluated regarding the levels of 145 microRNAs in the serum [28]. In the present study, approximately 30 samples were available for the interaction analyses with SIRT1, and 126 microRNA were analyzed.

### 2.5. Statistical Methods

The descriptive data are presented as percentages or mean ± standard deviation (SD). For continuous variables, a Student’s unpaired two-sided *t*-test was used, and the chi-square test was used for the analysis of one discrete variable. A slight non-Gaussian distribution of the dataset could be seen, and therefore, the dataset was log-transformed when evaluating continuous variables to obtain a normal distribution. The effect of this transformation was controlled through a Kolmogorov–Smirnov test. Therefore, transformed data were used in the *t*-test evaluations. All evaluations were performed according to the intention-to-treat principle. Repeated measures of variance were used to assess individual changes in the concentrations of SIRT1. In the analysis of covariance (ANCOVA), both transformed and non-transformed data were applied, with no significant difference in the results. In the multivariable model, the SIRT1 level after 48 months was used as the dependent variable. Adjustments were made for age, C-reactive protein (CRP) fold change, SIRT1 concentration at inclusion, smoking, gender, hypertension, diabetes, ischemic heart disease (IHD) and active treatment. In the correlation analyses between certain circulating microRNAs and serum SIRT1 concentration, non-parametric correlation methods were applied (Spearman RhO). *p*-values < 0.05 were considered statistically significant, based on a two-sided evaluation. All data were analyzed using standard software (Statistica v. 13.2, Dell Inc., Tulsa, OK, USA).

## 3. Results

The baseline characteristics of the study population, divided into an intervention with active substances group and a placebo group, are shown in Table 1. The mean age of the total population was 76 years, and 48.8% of it were females. At inclusion, no significant differences in the clinical characteristics were observed between the randomized groups. No participants presented with NYHA functional class IV, which indicates symptoms also at rest. The plasma Se concentration at inclusion was below the required amounts (~110 μg/L) for the optimal expression of selenoproteins [30,31] and did not differ between the randomized groups, with mean (SD) of 67.4 (14.2) μg/L for the active treatment group and 67.2 (13.2) μg/L for the placebo group, *p* = 0.80). At baseline, SIRT1 was inversely correlated with the inflammatory markers CRP (r = −0.43, *p* < 0.001), P-selectin (r = −0.11, *p* = 0.042) and osteopontin (r = −0.30, *p* = 0.02). We also found a significant correlation between the von Willebrand factor, a biomarker also for endothelial function, and SIRT1 (r = 0.52, *p* = 0.01). However, no significant correlation between Se concentration and levels of SIRT1 could be found at baseline (*p* > 0.05).

### 3.1. SIRT1 Concentration in Relation to the Se and Coenzyme Q_10_ Intervention

**Figure 2:**
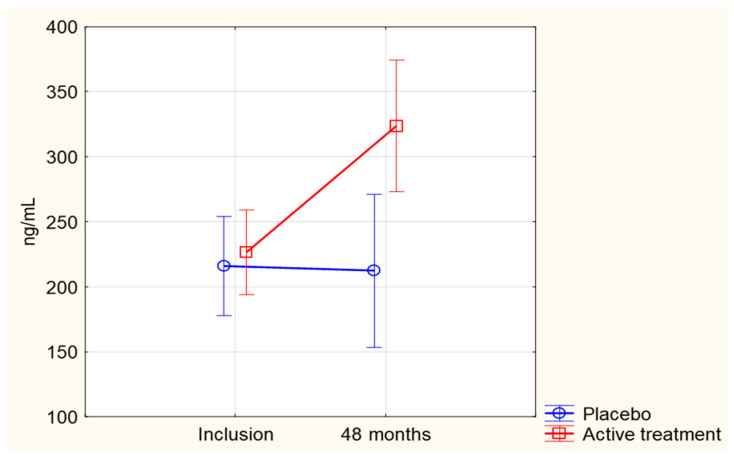
Concentration of SIRT1 at inclusion and after 48 months in the Se and coenzyme Q_10_ treatment group compared to the placebo group. Evaluation performed by use of the repeated measures of variance methodology. Current effect: F(1, 132) = 4.7026, *p* = 0.03. Vertical bars denote 0.95 confidence intervals. Blue curve: Placebo; Red curve: Active treatment group.

No significant difference in SIRT1 levels between the active treatment group and the placebo group was observed at baseline, with mean (SD) of 252 (162) ng/mL vs. 269 (172) ng/mL, *p* = 0.36. After the intervention, the SIRT1 levels increased significantly in the active treatment group, reaching 469 (436) ng/mL, *p* < 0.001), whereas the SIRT1 levels decreased in the placebo group to 190 (186) ng/mL, *p* = 0.002. The difference in change from baseline to 48 months between the two randomized groups was significant (*p* = 0.03, applying repeated measures of variance) (Figure 2).

When adjusting for age, sex, hypertension, smoking, diabetes, IHD, change in CRP levels and SIRT1 level at baseline in a multivariable model, significantly higher SIRT1 levels could still be observed at 48 months in the active group compared with the placebo group, (*p* = 0.006, Table 2).

### 3.2. SIRT1 Changes as Related to CV Mortality

**Figure 3:**
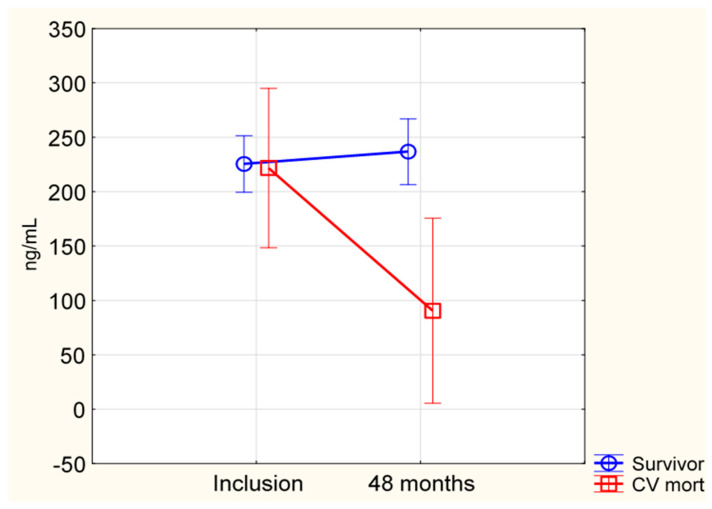
Concentration of SIRT1 in the total study population at inclusion and after 48 months, divided in the CV mortality and survivor groups, reported up to 10 years after inclusion. Evaluation performed by use of the repeated measures of variance methodology. Current effect: F(1, 132) = 5.9471, *p* = 0.016. Vertical bars denote 0.95 confidence intervals. Blue curve: Survivor; Red curve: CV mortality group.

Ten years after the study, a total of 77 CV deaths were registered, 25 (15%) in the active treatment group and 52 (32%) in the placebo group (*p* < 0.001). At the study start, the SIRT1 levels were already higher in the group that ended up as CV survivors compared to that of CV deaths, with mean (SD) of 319 (209) ng/mL vs. 242 (148), *p* < 0.001. To take into account the individual change in SIRT1 concentration, repeated measures of variance were applied to the study population divided into survivors and those who suffered CV death during a follow-up time of 10 years. We observed that the significantly higher SIRT1 levels in the survivor group persisted and that the difference in this change between the two randomized groups was significant (*p* = 0.016) (Figure 3).

Then, we evaluated the active treatment group and placebo group separately. In the placebo group, significantly lower concentrations of SIRT1 were observed in those who suffered CV mortality than in the survivors, i.e., 59 (33) ng/mL vs. 196 (164) ng/mL, *p* = 0.01. In the active treatment group, the SIRT1 concentration in the survivor group was higher than in the CV mortality group, i.e., 263 (178) ng/mL vs. 153 (54) ng/mL; however, these figures were not significantly different (*p* = 0.17). The lack of statistical difference was probably due to a highly restricted sample size in this sub-analysis.

### 3.3. Association between Circulating SIRT1 and microRNAs at Baseline

Of the 126 analyzed microRNA, 9 were significantly associated with SIRT1, of which 5 were inversely correlated (Table 3). Three of these microRNAs (bolded) have previously been reported to target SIRT1.

## 4. Discussion

The main finding of this investigation is that a four-year intervention with combined Se and coenzyme Q_10_ significantly increased the serum concentration of SIRT1, and this elevation was associated with reduced CV mortality. This is, to the best of our knowledge, the first time such an influence on SIRT1 has been reported in a clinical setting. We suggest that the observed increase in SIRT1 operates as a mediator and thus contributes to protection against vascular ageing and atherosclerosis. The mechanisms of SIRT1 elevation and protective actions are discussed in the following paragraphs.

### 4.1. Effects of the Se/CoQ10 Intervention on SIRT1

Se has multiple health-promoting properties, especially when given as a supplement to the elderly and in general to subjects with a low Se intake, the latter being frequently encountered in European countries due to the low Se concentration in the soil [1]. Dietary Se is incorporated into selenoproteins and, together with coenzyme Q_10_, these components play an important role in the body’s redox regulation and antioxidant defense. The beneficial effects of Se seem partly mediated by sirtuins, mainly by enhancing SIRT1 anti-inflammatory effects [2,19]. The observed significant and independent rise in SIRT1 may impact several biomolecules and signaling pathways. SIRT1 deacetylates multiple targets, including histones, leading to reduced transcription of the corresponding DNA, and non-histone proteins, including NF-κB, FOXO transcription factors, p-53, peroxisome proliferator-activated receptor-gamma coactivator (PCG)-1α and endothelial nitric oxide synthase (eNOS) among others. Hence, SIRT1 is implicated in multiple cellular processes such as metabolism, redox state, DNA transcription and repair, maintenance of genomic stability, apoptosis and organism lifespan [20,23,24,32]. Whether circulating SIRT1 reflects all these intracellular processes is not clear [24]. A possible rise in SIRT1 expression or circulating levels, and also its increased activity, does not necessarily reflect the same condition, and eventual differences also seem to be dependent on the type of tissue, organ, disease and the actual mechanism involved [33]. Increased compensating SIRT1 gene expression may also reflect low intracellular SIRT1 activity. To exemplify this controversy, high levels of SIRT1 concentration have been associated with non-alcoholic fatty liver disease, probably due to compensatory mechanisms [34]. We recently reported reduced circulating SIRT1 levels after bariatric surgery 6 and 12 months after the procedure, probably due to the loss of adipose tissue and consequently the reduced expression of SIRT1 mRNA in this site [35]. In the same study, we also showed that the levels of triglycerides were inversely predictive of SIRT1 levels. Recently, a meta-analysis of randomized controlled trials reported that coenzyme Q_10_ markedly reduced triglycerides, which again might explain the rise in SIRT1 after Se/Q10 supplementation [36]. Our group also previously showed that mRNA SIRT1 expression in circulating leukocytes was significantly reduced in type 1 diabetes patients, which was accompanied by elevated serum SIRT1 levels in diabetes patients with coronary heart disease compared to patients without this condition [37].

We believe that the rise in circulating SIRT1 concentrations in the present study is “genuine” due to the beneficial effects of the intervention.

### 4.2. Effects of SIRT1 in the CV System

SIRT1 seems also to be involved in cardiac metabolism and health, but the influence of Se on SIRT1 in CVD has been sparsely reported. However, recently, it was reported that SIRT1 has a major regulatory function in hypoxia-induced oxidative stress in cardiomyocytes [38]. Circulating SIRT1 was also previously shown to be reduced in elderly Italians with CVD in the presence of Se deficiency [19]. In addition, in a review, Packer et al. reported the cardioprotective effects of SIRT1, which would especially benefit heart failure patients [39], and Shengyu et al. reported a positive relationship between Se and the expression of SIRT1, evidencing a protective effect in cardiac hypertrophy [40]. Circulating SIRT1 was further reported to be inversely associated with epicardial fat thickness in obese subjects [41], and the plasma levels of SIRT1 were lower in patients with acute cerebrovascular stroke compared to controls, with no significant difference between ischemic and hemorrhagic groups [42]. Reduced levels were observed in pregnant women developing preeclampsia compared to women with healthy pregnancies, possibly due to increased oxidative stress, endothelial impairment and a reduction in SIRT1 expression [43].

We found that SIRT1 concentration at 48 months was higher in all survivors in comparison with the subjects in the CV mortality group, and the difference in the change from baseline between the groups was highly significant. The lower SIRT1 levels in those who suffered CV mortality within 10 years compared to those in the survivors at study inclusion could be explained by the fact that the negative development ending up with CV mortality had begun long before the study started.

### 4.3. Potential Mechanisms of Increased SIRT1 for Cardiac Protection

SIRT1 activity is regulated by NAD^+^ availability, and the NAD^+^ levels are thought to decrease with age [44]. Previous studies indicated that the Se/coenzyme Q_10_ intervention can increase the levels of NAD^+^. Se-methylselenocysteine, a naturally occurring organoselenium compound found in many plants and selenized yeast [45], was found to restore the NAD^+^ levels in human mammary epithelial cells when exposed to carcinogens [46]. The endogenous production of coenzyme Q_10_ also seems to decline with age, at least in some tissues [7,8,9]. With coenzyme Q_10_ deficit, the cytoplasmic and mitochondrial NAD^+^/NADH ratio is reduced. Coenzyme Q_10_ deficiency has also been found to lower SIRT1 mRNA expression [26]. Thus, a potential rise in NAD^+^ and the NAD^+^/NADH ratio by the Se and coenzyme Q_10_ intervention might have raised SIRT1 activity in CV cells, ensuing cardioprotective effects. SIRT1 is highly expressed in endothelial cells, although reduced in endothelial cells from older human arteries compared to those from younger adults [47]. Thus, the rise in SIRT1 after the intervention may have improved the vascular endothelial function via eNOS and increased NO production. The rise in SIRT1 may also have reduced the inflammatory signaling via NF-κB inhibition, resulting in reduced cytokine production in cardiac cells. The observed increase in genomic stability by our group, shown by the stabilization of telomeres after the Se/Coenzyme Q_10_ intervention [18], may also partly be mediated by SIRT1 through telomere reverse transcriptase induction [48]. With an elevation of the SIRT1 levels, a potential increased deacetylation of FOXO transcription factors and PGC-1α is thought to induce anti-oxidative enzymes, including glutathione peroxidase 1 (GPx1) and selenoprotein P (selenop) [49], thereby lowering cellular and extracellular oxidative stress [32]. We previously reported on the reduced levels of two oxidative stress biomarkers, adrenomedullin (MR-proADM) and copeptin, as an effect of the Se/Q_10_ intervention [16], indicating its beneficial influence on redox regulation, with a potential impact on SIRT1 activity. Additionally, an elevated SIRT1 concentration may also suppress the formation of foam cells, as SIRT1 has the ability to reduce the uptake of oxidized LDL and increase the reverse cholesterol uptake, thereby preventing plaque progression [32].

### 4.4. Regulation of SIRT1 by microRNAs

The regulation of SIRT1 synthesis may also include epigenetic regulation via microRNAs [50]. Recently, an increase in miR-130 was reported to affect SIRT1 mRNA in organismal and skin ageing [51]. In the study of microRNAs published by Alehagen et al. [28], the baseline Se concentration was observed to be inversely correlated with the expression of miR-130a-3p, and miR-130a-3p was significantly downregulated in the active treatment group compared to the placebo group at 48 months after the intervention. We observed that miR-130a-3p was significantly and inversely associated with SIRT1 at inclusion, suggesting that a decrease in this microRNA upon intervention might have contributed to the observed rise in SIRT1. MiR-222-3p has also recently been reported to target SIRT1 in cancer, arthritis and non-alcoholic fatty liver disease [52,53,54]. We observed that SIRT1 was also inversely correlated with miR-122-3p at baseline; however, the influence of the intervention on this relationship is less clear, as miR-122-3p was observed upregulated at 48 months [28]. MiR-181, defined to be a target of SIRT1 [29], was positively correlated with SIRT1 but was not markedly changed after the intervention, which is somewhat difficult to explain. That said, we cannot exclude a false observation due to the limited number of samples. In CVD, miR-199,has been shown to be upregulated in states of hypoxia with association to atherosclerosis and heart failure [50]. The previously reported SIRT1 downregulation during acute ischemia [24] might involve such SIRT1 regulation. We have previously reported that miR-199 was one of the most downregulated microRNAs after four years of Se/coenzyme Q_10_ supplementation [28], which also could have contributed to the rise in SIRT1 concentration, although no significant association with SIRT1 was observed. The miRs miR-34 and miR-133 known to target SIRT1 were also not associated with SIRT1 in the present study.

### 4.5. Limitations

Although highly significant and clear results were achieved overall, the limited number of participants in the sub-group analyses may have been inadequate, and therefore we may have failed to detect potential associations (statistical error Type II). Another limitation is the lack of SIRT1 mRNA measurements, which could have strengthened our results. As RNA sampling was not performed initially, SIRT1 gene expression analysis could unfortunately not be accomplished. Except for the interaction between SIRT1 and miR-130a-3p, which underlines potential mechanisms of the Se/coenzyme Q_10_ intervention, the associations between SIRT1 and other investigated microRNAs in this context warrant further investigation.

In addition, the differential influence of Se and coenzyme Q_10_ on SIRT1 was not possible to be explored in the present study.

## 5. Conclusions

After four years of supplementation with combined Se and coenzyme Q_10_, we found SIRT1 concentration to be significantly increased, which was potentially mediated by miR-130a-3p downregulation, among other microRNAs, ensuing CV protection with a significant reduction in CV mortality. The importance of Se and coenzyme Q_10_ in the prevention of CVD and the role of SIRT1 in this context highlight the beneficial effects of SIRT1 on CV functions, suggesting SIRT as a target for potential prevention.

## Figures and Tables

**Table 1:** Baseline characteristics of the participants divided into an active supplementation group and a placebo group.

	Active Treatment Group *n* = 165	Placebo Group *n* = 161	*p*-Value
Age years	76.1 (3.2)	76.4 (3.0)	0.43
Sex, Males/Females n	85/80	82/79	
History			
Smoking, n (%)	8 (4.8)	8 (5.0)	0.96
Diabetes, n (%)	34 (20.6)	29 (18.0)	0.55
Hypertension, n (%)	118 (71.5)	116 (72.0)	0.91
IHD, n (%)	25 (15.2)	29 (18.0)	0.49
Atrial fibrillation, n (%)	14 (8.5)	16 (9.9)	0.65
NYHA class I, n (%)	99 (60.0)	87 (54.0)	0.28
NYHA class II, n (%)	44 (26.7)	44 (27.3)	0.89
NYHA class III, n (%)	21 (12.7)	28 (17.4)	0.24
NYHA class IV, n (%)	0	0	
Unclassified, n (%)	1 (0.6)	2 (1.2)	
Medications			
ACEI/ARB, n (%)	28 (17.0)	34 (21.1)	0.34
Beta blockers, n (%)	55 (33.3)	46 (28.6)	0.35
Diuretics, n (%)	46 (27.9)	60 (37.3)	0.07
Statins, n (%)	32 (19.4)	37 (23.0)	0.73
Examinations			
EF < 40%, n (%)	7 (4.2)	6 (3.7)	0.81
s-selenium s-coenzyme Q_10_	67.4 (14.2) 0.84 (0.31)	67.2 (13.2) 0.88 (0.34)	0.80 0.68
CV-death, n (%)	25 (15)	52 (32)	<0.001

ACEI: ACE inhibitors; ARB: Angiotensin receptor blockers; EF: Ejection fraction; IHD: Ischemic heart disease, NYHA: New York Heart Association functional class; SD: Standard Deviation. Values are means ± SD or frequency (percent). The Student’s unpaired two-sided *t*-test was used for continuous variables, and the chi-square test was used for the analysis of one discrete variable.

**Table 2:** Analysis of covariance using SIRT1 level after 48 months as the dependent variable.

Effects	Sum of Squares	Degrees of Freedom	Mean Squares	F	*p*
Intercept	389,335	1	389,335	14.37	0.0002
Age >75 years	4755	1	4755	0.18	0.68
CRP fold change	77,029	1	77,029	2.84	0.09
SIRT1 baseline	13,786	1	13,786	0.51	0.48
Smoker	2494	1	2494	0.09	0.76
Male sex	18,523	1	18,523	0.68	0.41
Hypertension	86,222	1	86,222	3.18	0.08
Diabetes	5233	1	5233	0.19	0.66
IHD	35,406	1	35,406	1.31	0.26
Active treatment	210,429	1	210,429	7.77	**0.006**
Error	3,115,998	115	27,095		

CRP: C-reactive protein, IHD: Ischemic heart disease. Bold *p*-value indicates statistical significance of the effect of the active treatment.

**Table 3:** Significant correlations ^1^ between microRNAs and SIRT1.

MicroRNAs	*n*	*r*	*p*
**miR-130a-3p**	**32**	***r* = −0.466**	**0.007**
miR-19b-3p	27	*r* = −0.447	0.019
miR-16-2-3p	25	*r* = −0.459	0.020
**miR-222-3p**	**27**	***r* = −0.438**	**0.022**
miR-454-3p	22	*r* = −0.446	0.040
miR-423-3p	27	*r* = 0.535	0.004
miR-30b-5p	27	*r* = 0.523	0.005
**miR-181-5p**	**27**	***r* = 0.488**	**0.010**
miR-191-5p	27	*r* = 0.393	0.042

^1^ Spearman Rho correlation. miR: microRNA. Bold text indicates microRNAs known to target SIRT1.

## Data Availability

Research data can be available on reasonable request to the last author.
